# Effects of the Cessation of Mass Screening for Neuroblastoma at 6 Months of Age: A Population-Based Study in Osaka, Japan

**DOI:** 10.2188/jea.JE20150054

**Published:** 2016-04-05

**Authors:** Akiko Ioka, Masami Inoue, Akihiro Yoneda, Tetsuro Nakamura, Junichi Hara, Yoshiko Hashii, Naoki Sakata, Kazumi Yamato, Hideaki Tsukuma, Keisei Kawa

**Affiliations:** 1 Center for Cancer Control and Statistics, Osaka Medical Center for Cancer and Cardiovascular Diseases, Osaka, Japan; 2 Department of Hematology/Oncology, Osaka Medical Center and Research Institute for Maternal and Child Health, Izumi, Osaka, Japan; 3 Department of Pediatric Surgery, Children's Medical Center, Osaka City General Hospital, Osaka, Japan; 4 Children's Medical Center, Osaka City General Hospital, Osaka, Japan; 5 Department of Pediatrics, Osaka University Graduate School of Medicine, Suita, Osaka, Japan; 6 Department of Pediatrics, Kinki University School of Medicine, Osakasayama, Osaka, Japan; 7 Department of Pediatrics, Osaka City University Graduate School of Medicine, Osaka, Japan

**Keywords:** neuroblastoma, cessation, screening, mortality, incidence

## Abstract

**Background:**

In 2004, the Japanese government halted the 6-month mass screening program for neuroblastoma. We investigated whether its cessation had led to an increase not only in mortality due to this disease but also in the incidence of advanced-stage disease among older children.

**Methods:**

Study subjects were neuroblastoma patients retrieved from the population-based Osaka Cancer Registry. Trends of incidence and mortality from neuroblastoma were analyzed by calendar year and birth cohort. Prognostic factors, including stage and v-myc avian myelocytomatosis viral oncogene neuroblastoma derived homolog (MYCN) oncogene status, were compared before and after the cessation of mass screening.

**Results:**

Age-standardized incidence rates in 2005–2009 (the cessation period of mass screening; 11.1 per million) were similar to those in 1975–1979 (the pre-screening period; 8.6 per million). Age-standardized mortality rates tended to decrease from 1975–1979 (4.0 per million) to 2005–2009 (2.7 per million) in parallel with the improvement in survival. Analysis by birth cohort indicated that the mortality rates in 2004–2005 (after cessation) for children 0–4 years of age were lower than those in 1975–1979 (O:E ratio 0.25; 95% confidence interval, 0.03–0.90). For children 1–9 years of age, there was a not significant difference in the distribution of stage, MYCN oncogene status, and DNA ploidy between 1991–2003 (the mass screening period) and 2004–2008 (after cessation).

**Conclusions:**

The cessation of mass screening for neuroblastoma does not appear to have increased mortality due to this disease or incidence of advanced-stage disease among older children.

## INTRODUCTION

Neuroblastoma is the second most common extracranial solid tumor in children under the age of 15 years.^1^ Nationwide mass screening for neuroblastoma at 6 months of age has been performed in Japan since 1985. Previous studies^2^^–^^7^ showed that routine screening for neuroblastoma did not reduce the incidence of disseminated disease or mortality due to this disease. In 2004, the Japanese government^8^ halted the mass screening program, as it tended to over-diagnose localized tumors with favorable prognoses,^9^^–^^11^ including occult tumors that regressed spontaneously or matured without ever becoming clinically apparent, and did not reduce mortality due to neuroblastoma. After the cessation of the routine screening program, however, neither population-based neuroblastoma mortality nor advanced-stage incidence has been assessed in Japan.

In Japan, the Osaka Cancer Registry (OCR) covers the largest population among the population-based registries^12^; there is no registry that covers the entire Japanese population. Therefore, we used OCR data to assess whether the cessation of the 6-month mass screening program for neuroblastoma has led to an increase not only in disease mortality but also in advanced-stage disease incidence, especially among older children.

## METHODS

### Screening in Osaka

In Osaka, the mass screening program for neuroblastoma at 6 months of age by qualitative assessment of urinary vanillylmandelic acid (VMA) started in 1985, and assessment by quantitative high-performance liquid chromatography (HPLC) began in 1988. The screening consisted of testing urine to determine the levels of catecholamine metabolites, VMA, and homovanillic acid, which are elevated in clinically presenting neuroblastoma. Of all live births in Osaka, 2%–29% were screened during the period of partial VMA assessment (1980–1984), 57%–61% were screened during the period of nearly complete VMA assessment (1985–1987), and 68%–81% during the period of nearly complete HPLC (1988–2003). In 2004, the Japanese government stopped the screening program.

### Data sources

A total of 662 reported cases of ganglioneuroblastoma and neuroblastoma (morphology codes 9490/3 and 9500/3 in the International Classification of Diseases for Oncology, third edition) newly diagnosed in 1975–2009 were retrieved from the OCR database. OCR has been operating since December 1962 and covers the entire population of Osaka Prefecture (8.8 million according to the 2010 census). Cancer incidence data in Osaka have been reported in ‘Cancer Incidence in Five Continents’ from volume III in 1976 to X in 2013,^12^ and in ‘International Incidence of Childhood Cancer’ from volume II in 1998^13^ to volume III, which is forthcoming. Therefore, it can be assumed that the quality of these data for both all cancer and childhood cancer meets the standards set by the International Association of Cancer Registries in the last four decades. Among our study subjects, the proportions of death-certificate-only (DCO) cases in 0-, 1- to 4-, 5- to 9-, and 10- to 14-year-olds were 0.0%, 0.8%, 0.0%, and 0.0%, respectively; the proportions of DCO cases in 1975–1979, 1980–1984, 1985–1989, 1990–1994, 1995–1999, 2000–2004, and 2005–2009 diagnosis periods were 0.0%, 1.7%, 0.9%, 0.0%, 0.0%, 0.0%, and 0.0%, respectively.

To calculate mortality rates, we used data for 201 cases of ganglioneuroblastoma or neuroblastoma who died between 1975 and 2009 and who were under 15 years of age at death. Based on the quality of data collection on the vital status of registered cases, survival analysis was restricted to those who lived in Osaka Prefecture (except for Osaka City) in 1975–2007 or resided in Osaka City in 1993–2007 when they were diagnosed with ganglioneuroblastoma or neuroblastoma, since active follow-up information was available for these cases. For cases with multiple tumors, only the first was included. In total, 14 cases (2.7%) were lost to follow-up, which meant their vital status could not be determined by referencing the basic resident register, and were treated as censored cases at the latest date when they were confirmed to be alive.

We identified 334 patients diagnosed with ganglioneuroblastoma or neuroblastoma during 1991–2008, and sent questionnaires to major hospitals in Osaka to obtain information on prognostic factors^14^^–^^19^ (stage and status of v-myc avian myelocytomatosis viral oncogene neuroblastoma derived homolog [MYCN] and DNA ploidy status). Additional clinical data for 268 cases was assembled, which covered 80.2% of all study subjects: coverage was 79.3% in 1991–2003 (the HPLC mass screening period) and 88.2% in 2004–2008 (after cessation of mass screening). The stage was classified according to the International Neuroblastoma Staging System (INSS) definition.^20^^–^^23^

This study design was approved by the ethics review boards of the Osaka Medical Center for Cancer and Cardiovascular Diseases (Osaka, Japan) in 2012.

### Statistical analysis

The population under 15 years of age was estimated using linear interpolation from census data for every 5 years during the period 1975–2009. Age-standardized rates were calculated using the world population for the four age groups of 0, 1–4, 5–9, and 10–14 years. The standardized rate ratio of age-standardized rates between those in 1975–1979 (unscreened) and those in 1980–2004 (partial or systematic screening)/2005–2009 (after cessation of screening) were calculated with 95% confidence intervals.

A birth cohort analysis was conducted to clarify the effects of each method of mass screening on the changes in age-specific incidence and mortality rates. Years of birth were divided into five groups: 1975–1979 (unscreened), 1980–1984 (partial VMA), 1985–1987 (nearly complete VMA), 1988–2003 (nearly complete HPLC), and 2004–2005 (after cessation of mass screening).

Cumulative observed survival was estimated using the Kaplan-Meier method according to age groups. Survival time was computed from the date of first diagnosis to the end-point, defined as death from any cause. Closing date was defined as the date 5 years after the first diagnosis. Relative survival was calculated as the ratio of observed survival to expected survival (O:E ratio); the latter was estimated using the probability of survival in the general population of Japan for similar subjects with respect to sex, age by 1-year increments (0–99 years old) and calendar year at diagnosis (National Cancer Center, http://ganjoho.jp/professional/statistics/cohort01.html). Relative survival was evaluated with *Z*-tests^24^ compared with cases in 1975–1984. The distributions of patients’ prognostic factors were assessed with Fisher’s exact tests for categorical variables; distributions in the stage, MYCN status, and DNA ploidy were calculated among all cases, excluding “undone” or “unknown”. Differences were considered statistically significant if *P* values were less than 0.05 by two-sided test. Data management and statistical analyses were conducted with STATA (StataCorp, College Station, TX, USA).^25^

## RESULTS

The age-standardized incidence and mortality rates (per million children) of neuroblastoma in 1975–2009 were 7.5–24.4 (incidence rate) and 2.7–5.5 (mortality rate) (Table 1). The incidence rate increased remarkably from 1975–1979 to 1985–1989, was almost stable through 1985–2004, and then decreased in 2005–2009; the incidence rate in 1985–2004 increased significantly to about twice that of 1975–1979. The mortality rate increased in 1980–1989 as compared with that in 1975–1979, and tended to decrease during the last period. The increase in incidence rate was remarkable for those under 1 year of age: the rates per million children under 1 year of age between 1985–1989 and 2000–2004 were 4–8 times as high as that in 1975–1979. The incidence rate at more than 1 year of age did not change during these periods.

**Table 1.** Number of patients and annual rate per million children of neuroblastoma for both sexes in Osaka, 1975–2009

	Period	Number of cases	Age-specific rate				ASR	SIR/SMR (95% CI)	

			0	1–4	5–9	10–14			
Incidence	1975–1979	84	27.7	16.7	3.5	0.6	8.6	1.0	
	1980–1984	60	24.1	14.6	3.2	0.6	7.5	0.9	0.8 to 1.0
	1985–1989	111	109.7	21.8	3.7	1.8	16.7	1.9	1.8 to 2.1
	1990–1994	141	220.6	18.3	5.2	0.8	24.4	2.8	2.6 to 3.1
	1995–1999	99	164.6	12.5	2.9	0.4	17.9	2.1	1.9 to 2.3
	2000–2004	111	139.3	22.2	7.3	1.0	20.2	2.3	2.1 to 2.6
	2005–2009	56	27.5	26.4	2.5	0.0	11.1	1.3	1.2 to 1.4
Mortality	1975–1979	39	4.4	9.7	1.6	0.3	4.0	1.0	
	1980–1984	44	11.1	12.0	2.3	0.6	5.5	1.4	1.2 to 1.6
	1985–1989	38	10.5	11.2	2.2	1.5	5.4	1.4	1.2 to 1.6
	1990–1994	27	9.3	10.3	1.7	0.4	4.6	1.2	1.0 to 1.3
	1995–1999	24	7.0	8.9	2.4	0.4	4.2	1.1	0.9 to 1.2
	2000–2004	15	7.3	6.0	1.0	0.0	2.7	0.7	0.6 to 0.8
	2005–2009	14	0.0	7.7	1.0	0.0	2.7	0.7	0.6 to 0.8

ASR, age-standardized rate to the world population; CI, confidence interval; SIR/SMR, standardized incidence rate/standardized mortality rate.

Table 2 presents the results of the birth cohort analysis. Incidence per 100 000 live births significantly increased at 0 years of age following the introduction of mass screening, especially by HPLC: the incidence rate at 0 years of age per 100 000 live births increased from 3.01 in 1975–1979 to 17.61 in 1988–2003 and also significantly increased from 3.16 to 4.87 at 2–4 years of age. The O:E ratio of those born in 1988–2003 compared to those born in 1975–1979 was 5.84 at 0 years of age and 1.54 at 2–4 years of age. The mortality rate in 1988–2003 for both 0 and 2–4 years of age was slightly lower than that in 1975–1979 (O:E ratios 0.95 and 0.70; 95% confidence intervals, 0.46–1.75 and 0.47–1.01, respectively), and also those in 2004–2005 (O:E ratios 0.00 and 0.22; 95% confidence intervals, 0.00–3.14 and 0.01–1.25, respectively).

**Table 2.** Incidence and mortality of neuroblastoma by birth cohort in Osaka, 1975–2009

Method of screening	Age, years	Incidence				Mortality			
	
		*n*	Rate^b^	O:E (95% CI)		*n*	Rate	O:E^c^ (95% CI)	
Unscreened (1975–79) [663 558]^a^	0–4	61	9.19	1.00		34	5.12	1.00	
	1–4	41	6.18	1.00		29	4.37	1.00	
	2–4	21	3.16	1.00		19	2.86	1.00	
	1	20	3.01	1.00		10	1.51	1.00	
	0	20	3.01	1.00		5	0.75	1.00	

Partial VMA (1980–84) [533 537]	0–4	58	10.87	1.18	0.90 to 1.53	35	6.56	1.28	0.89 to 1.78
	1–4	42	7.87	1.27	0.92 to 1.72	29	5.44	1.24	0.83 to 1.79
	2–4	23	4.31	1.36	0.86 to 2.04	19	3.56	1.24	0.75 to 1.94
	1	19	3.56	1.18	0.71 to 1.85	10	1.87	1.24	0.60 to 2.29
	0	16	3.00	1.00	0.57 to 1.62	6	1.12	1.49	0.55 to 3.25

Nearly complete VMA (1985–1987) [292 849]	0–4	43	14.68	1.60	1.16 to 2.15	17	5.81	1.13	0.66 to 1.81
	1–4	21	7.17	1.16	0.72 to 1.77	13	4.44	1.02	0.54 to 1.74
	2–4	14	4.78	1.51	0.83 to 2.53	9	3.07	1.07	0.49 to 2.04
	1	7	2.39	0.79	0.32 to 1.63	4	1.37	0.91	0.25 to 2.32
	0	22	7.51	2.49	1.56 to 3.77	4	1.37	1.81	0.49 to 4.64

Nearly complete HPLC (1988–2003) [1 396 812]	0–4	355	25.42	2.77	2.48 to 3.07	51	3.65	0.71	0.53 to 0.94
	1–4	109	7.80	1.26	1.04 to 1.52	41	2.94	0.67	0.48 to 0.91
	2–4	68	4.87	1.54	1.19 to 1.95	28	2.00	0.70	0.47 to 1.01
	1	41	2.94	0.97	0.70 to 1.32	13	0.93	0.62	0.33 to 1.06
	0	246	17.61	5.84	5.14 to 6.62	10	0.72	0.95	0.46 to 1.75

Cessation of screening (2004–2005) [155 830]	0–4	13	8.34	0.91	0.48 to 1.55	2	1.28	0.25	0.03 to 0.90
	1–4	7	4.49	0.73	0.29 to 1.50	2	1.28	0.29	0.04 to 1.06
	2–4	2	1.28	0.41	0.05 to 1.46	1	0.64	0.22	0.01 to 1.25
	1	5	3.21	1.07	0.35 to 2.48	1	0.32	0.43	0.01 to 2.37
	0	6	3.85	1.28	0.47 to 2.78	0	0.00	0.00	0.00 to 3.14

CI, confidence interval; O:E, Observed:Expected ratio.

^a^Number of live births during the period.

^b^Rate per 100 000 live births.

^c^The number of expected cases was calculated from the age-specific rate in 1975–1979.

Relative 5-year survival for neuroblastoma in under-15-year-olds significantly improved from 36.7% (standard error [SE] 5.1%) in 1975–1984 to 82.5% (SE 5.0%) in 2004–2007 (Table 3). There were significant improvements in survival for both 0–1 years and 2–14 years of age: the survival for 2–14 years of age dramatically increased from 15.7% (SE 5.5%) to 77.2% (SE 7.1%).

**Table 3.** Relative 5-year survival for neuroblastoma cases

	0–14 years old			0–1 year old			2–14 years old		
		
	*n*	Relative 5-year survival	*P* value^b^	*n*	Relative 5-year survival	*P* value	*n*	Relative 5-year survival	*P* value
Total	525 (100.0)	72.3 (2.0)^a^		343 (100.0)	85.6 (1.9)		182 (100.0)	47.6 (3.7)	
Year of diagnosis									
1975–1984	97 (18.5)	36.7 (5.1)	—	49 (14.3)	57.2 (7.3)	—	48 (26.4)	15.7 (5.5)	—
1985–2003	368 (70.1)	79.6 (2.1)	<0.01	269 (78.4)	90.1 (1.9)	<0.01	99 (54.4)	51.6 (5.0)	<0.01
2004–2007	60 (11.4)	82.5 (5.0)	<0.01	25 (7.3)	91.0 (6.1)	<0.01	35 (19.2)	77.2 (7.1)	<0.01

^a^Figures in parentheses are standard errors.

^b^Relative 5-year survival was evaluated as compared with cases in 1975–1984.

Table 4 shows the prognostic factors for neuroblastoma cases. Their distributions were compared before and after cessation of mass screening. There was a significant difference in the distribution of stage for 0 years of age between 1991–2003 and 2004–2008, and the proportion of stage 1/2A/2B cases tended to be higher among the former period (69.2% vs 36.4%). The distribution of the MYCN status and DNA ploidy did not change significantly for any age group during these periods. Although not significant, the proportion of non-amplified MYCN or non-diploid subjects was higher than in subjects with amplified MYCN or diploid subjects, which seemed to be associated with the proportion of subjects with stage 1/2A/2B disease at 0 years of age being higher than subjects with stage 3/4/4S disease.

**Table 4.** Prognostic factors for neuroblastoma cases

	1991–2003						2004–2008								
	
	0 year old		1–4 years old		5–9 years old		0 year old			1–4 years old			5–9 years old		
					
	*n*	%	*n*	%	*n*	%	*n*	%	*P* value^b^	*n*	%	*P* value	*n*	%	*P* value
Total	185	(100.0)	29	(100.0)	2	(100.0)	12	(100.0)		37	(100.0)		3	(100.0)	

Stage (INSS)									<0.05			0.44			0.40
1/2A/2B	128	(69.2)^a^	8	(27.6)	1	(50.0)	4	(36.4)		14	(37.8)		0	(0.0)	
3/4/4S	57	(30.8)	21	(72.4)	1	(50.0)	7	(63.6)		23	(62.2)		3	(100.0)	
Unknown	0	—	0	—	0	—	1	—		0	—		0	—	

MYCN									0.43			0.18			1.00
Amplified	7	(5.0)	7	(26.9)	1	(50.0)	1	(10.0)		16	(45.7)		1	(33.3)	
Non-amplified	134	(95.0)	19	(73.1)	1	(50.0)	9	(90.0)		19	(54.3)		2	(66.7)	
Undone	43	—	3	—	0	—	2	—		1	—		0	—	
Unknown	1	—	0	—	0	—	0	—		1	—		0	—	

DNA Ploidy									0.45			0.44			—
Diploid	30	(34.1)	7	(63.6)	2	(100.0)	4	(50.0)		20	(76.9)		2	(100.0)	
Aneuploid/Tetraploid	58	(65.9)	4	(36.4)	0	(0.0)	4	(50.0)		6	(23.1)		0	(0.0)	
Undone	71	—	12	—	0	—	2	—		6	—		0	—	
Unknown	26	—	6	—	0	—	2	—		5	—		1	—	

INSS, International Neuroblastoma Staging System; MYCN, v-myc avian myelocytomatosis viral oncogene neuroblastoma derived homolog.

^a^Figures in parentheses are proportions among total number of patients, excluding “undone” or “unknown”.

^b^Proportion was evaluated as compared with cases in 1991–2003.

## DISCUSSION

Well-known studies^2^^–^^6^ have reported that there was no reduction in the incidence of advanced disease or in mortality from neuroblastoma due to mass screening at 1 year of age or younger. However, the effects of the cessation of the mass screening on incidence and mortality have not been examined in a population-based study, and the purpose of our study was to clarify these effects in Osaka, Japan.

The incidence rate increased obviously in both the qualitative screening period and the quantitative screening period and then decreased after cessation. The mortality rate in the qualitative screening period was higher than that in pre-screening period but not that in the quantitative screening period and after cessation. These findings suggest that the higher mortality rate in the qualitative screening period might be due to the labeling effect of screening, as quantitative HPLC measurement can identify cases of neuroblastoma more accurately than the qualitative method. These results indicate that the mass screening affected the incidence and mortality; however, the findings did not support the usefulness of mass screening for neuroblastoma.

A significant decrease in mortality at 0–4 years of age was observed in the period of mass screening with quantitative assessment by HPLC, as well as after screening cessation, compared with the pre-screening period. Previous studies^2^^,^^4^^–^^6^ have reported that screening for neuroblastoma had no effect on mortality due to this disease. Ajiki et al^3^ suggested the reduction in mortality during the mass screening period was due to recent advances in treatment that improved survival for neuroblastoma patients rather than the screening process. Our results show mortality trends in the screening periods that are consistent with these reports, and the lower mortality rate remained stable after the cessation of screening, with significant improvement in the prognosis of patients diagnosed with neuroblastoma at 0–14 years of age in 1985–2003 and 2004–2007 compared to 1975–1984. Such a reduction in mortality could not be due to the effects of mass screening, as survival improved even after cessation of screening. Improvement in 5-year survival for neuroblastoma was also observed in the United States and Northern Europe during this decade.^26^^,^^27^

Age, stage, MYCN oncogene status, and DNA ploidy status were clinically relevant factors: unfavorable biologic features are older age, advanced stage, and amplified MYCN and diploid.^14^^–^^16^ The age-specific incidence rate for neuroblastoma under 1 year of age rapidly increased with the introduction of the screening program at 6 months of age according to calendar year analysis as well as birth cohort analysis, and the disease was more localized at a younger age, which is associated with markers of good prognosis. The proportion of stage 1/2A/2B tended to be higher among the group screened at 0 years of age than among the un-screened group and was associated with a lower proportion of MYCN-amplified or diploid tumors. After screening cessation, the incidence rate decreased to the pre-screening level, and cessation did not increase the incidence of advanced disease: distribution of the advanced stage at 1 year or older did not change after cessation compared with that during the screening program with HPLC. Previous studies have reported that patients who were diagnosed with neuroblastoma detected by the mass screening had favorable outcomes and that there was the possibility of over-diagnosis.^10^^,^^11^ Our results were similar to these, and we also found no evidence of increasing incidence of metastatic disease among older patients after the cessation of the mass screening program.

As reported in our previous study, a limitation of this study is that the reporting of cancer diagnoses to the cancer registry by medical institutions was incomplete, because the proportion of DCO cases for all sites (except non-melanoma skin cancer) in OCR was higher than that in North American or northern/western European registries. In OCR, more than 10% of cases were DCO. Therefore, incidence rates for neuroblastoma might have been underestimated in our study. The proportions of prognostic factors among patients diagnosed in 1991–2003 might also have been underestimated as, in Japan, there is no obligation to keep clinical information in hospitals or clinics for more than 5 years after the year of diagnosis. It was therefore difficult to gather information, especially for cases where more than 10 years had elapsed since diagnosis. Another limitation is in the generalizability of these study findings, since this study was based on the OCR database only. It is necessary to ascertain from nationwide cancer registry data whether or not similar patterns exist in other areas in Japan.

Despite some limitations inherent in our study, the cessation of mass screening for neuroblastoma does not appear to have increased population-based mortality due to this disease. Our results also suggest that there were no differences in the proportions of prognostic factors or patients with advanced stage, whether patients were diagnosed with neuroblastoma during the mass screening period or not.
